# Coverage of lateral soft tissue defects with sartorius muscle flap after distal femoral replacement for malignant bone tumors

**DOI:** 10.1051/sicotj/2024025

**Published:** 2024-08-13

**Authors:** Naoki Minami, Shinji Tsukamoto, Takamasa Shimizu, Kanya Honoki, Hideo Hasegawa, Tomoya Masunaga, Akira Kido, Costantino Errani, Andreas F. Mavrogenis, Yasuhito Tanaka

**Affiliations:** 1 Department of Orthopaedic Surgery, Nara Medical University 840 Shijo-cho Kashihara City Nara 634-8521 Japan; 2 Department of Rehabilitation Medicine, Nara Medical University Nara Japan; 3 Department of Orthopaedic Oncology, IRCCS Rizzoli Institute Bologna Italy; 4 First Department of Orthopaedics, National and Kapodistrian University of Athens, School of Medicine Athens Greece

**Keywords:** Sartorius muscle flap, Bone tumor, Distal femur, Megaprosthesis, Infection, Soft tissue defect

## Abstract

*Introduction*: To prevent infection after limb-sparing surgery for primary malignant bone tumors, it is important to cover the megaprosthesis with muscle tissue that has sufficient blood flow. Coverage with a lateral gastrocnemius flap has been reported in cases of distal femoral replacement in which the vastus lateralis and vastus intermedius muscles have been resected; however, the risk of peroneal nerve palsy is reportedly high because the muscle flap passes near the peroneal head. This study was performed to examine the postoperative outcomes of patients with primary malignant bone tumors of the distal femur who underwent wide resection (including the vastus lateralis and vastus intermedius muscles) followed by reconstruction with a megaprosthesis and coverage of the lateral side of the prosthesis with a sartorius muscle flap. *Methods*: We retrospectively analyzed three patients who underwent reconstruction with a megaprosthesis after wide resection of a primary malignant bone tumor of the distal femur involving the vastus lateralis and vastus intermedius muscles and reconstruction of the soft tissue defect on the lateral side of the prosthesis with a sartorius muscle flap. *Results*: The average defect size was 6 × 13 cm, the average time required for a sartorius muscle flap was 100 min, and the average implant coverage was 93%. The average postoperative follow-up period was 35 months, during which no postoperative complications such as infection, skin necrosis, or nerve palsy occurred. *Discussion*: The distally based sartorius muscle flap is easy to elevate in the supine position, has minimal functional loss after harvesting, and has minimal risk of nerve palsy. It can be advocated as the first option for coverage of soft tissue defects lateral to distal femoral replacement.

## Introduction

The distal femur is a common site of primary malignant bone tumors such as osteosarcoma and Ewing sarcoma [1]. Limb-sparing surgery using a megaprosthesis or allograft is the standard treatment [2]. Complications of limb-sparing surgery using a megaprosthesis include infection, loosening, implant breakage, and nerve palsy [3–5]. Infection is a serious adverse event and usually requires debridement, antibiotics, implant retention, or two-stage revision [6]. In patients scheduled for postoperative chemotherapy, amputation may be chosen because of the need for early infection control [7]. Thus, prevention of infection is especially important in patients with primary malignant bone tumors [8].

**Figure 1:**
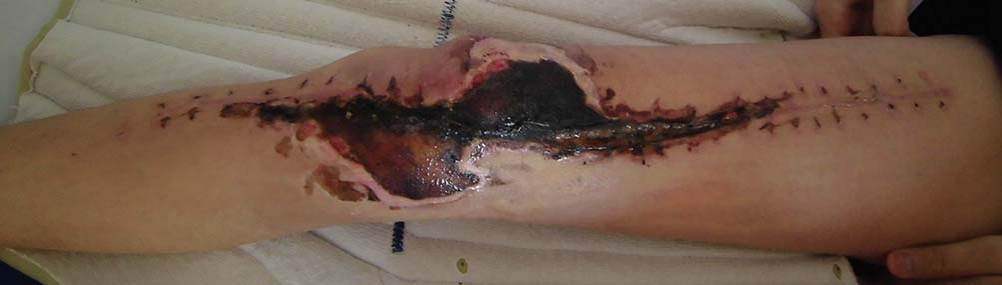
A patient with conventional osteosarcoma of the distal femur underwent reconstruction with a megaprosthesis after wide resection involving the vastus medialis, intermedius, and lateralis muscles. Skin necrosis developed, but the megaprosthesis was not covered by muscle, resulting in deep infection.

The factor most closely associated with the risk of uncontrolled infection after limb-sparing surgery leading to amputation is poor condition of the soft tissue [9–11]. In many cases, wide resection of a primary malignant bone tumor requires combined excision of muscles such as the quadriceps, extensive subcutaneous dissection, and excision around the incisional biopsy wound [12]. If the megaprosthesis is not covered by muscle, deep infection can develop secondary to skin necrosis [13, 14] (Figure 1). Therefore, it is important to cover the megaprosthesis with muscles that have sufficient blood flow to prevent infection [13, 14]. In cases where the vastus medialis and vastus intermedius muscles have been removed, covering the megaprosthesis with a medial gastrocnemius flap has been reported as the standard technique [15, 16]. In cases where the vastus lateralis and vastus intermedius muscles have been resected, coverage with a lateral gastrocnemius flap has been reported [12, 15]; however, the risk of peroneal nerve palsy is reportedly high (44%) because of the passage of the muscle flap near the peroneal head [12].

The sartorius muscle is a type III (Mathes and Nahai classification) elongated bifid muscle that originates from the anterior superior iliac spine (ASIS) and terminates on the medial surface of the proximal tibia [17, 18]. The sartorius muscle has a rich longitudinal vascular network running internally, and 80–90% of the muscle flap can reportedly be elevated with a single proximal or distal vascular pedicle [18]. Although sartorius muscle flaps utilizing distal vascular pedicles have been used for post-traumatic soft tissue defects around the knee [19], no reports have described their application to soft tissue reconstruction of megaprostheses following distal femoral resection. The sartorius muscle is superficial and easy to elevate in the supine position. Additionally, minimal functional loss occurs after harvesting, and the risk of nerve palsy is low [20]. We herein report a detailed surgical technique and its outcomes in three patients who underwent sartorius muscle flap coverage of a lateral soft tissue defect after distal femoral replacement for a malignant bone tumor.

## Materials and methods

We retrospectively analyzed three patients who underwent reconstruction with a megaprosthesis after wide resection of a primary malignant bone tumor of the distal femur (including the intermediate and lateral vastus muscles) and reconstruction of the soft tissue defect on the lateral side of the prosthesis with a sartorius muscle flap from 2020 to 2023 at our institution.

### Preparation for surgery

We determined the extent of resection with preoperative contrast-enhanced magnetic resonance imaging and contrast-enhanced computed tomography. We anticipated the location and size of the soft tissue defect and marked the vascular pedicles and pivot point of the sartorius muscle using preoperative ultrasound and contrast computed tomography [19].

### Surgical procedure

A clean tourniquet was used; it was subsequently removed if it interfered with the surgical procedures involving the proximal thigh. A skin incision was made with an approximately 2 cm margin around the incisional biopsy wound. The incisional biopsy scar was adherent to the tumor side and had developed with the fascia attached to the skin. The rectus femoris muscle was identified and elevated, and the patella was osteotomized for extra-articular resection. The vastus medialis, vastus intermedius, and vastus lateralis were resected along with the tumor, and the femur was osteotomized at the planned distance from the knee joint level. The femoral artery and vein and the tibial and peroneal nerves were detached while lifting the tumor, and the genicular arteries were ligated. The short head of the biceps femoris muscle and the attachment site of the medial and lateral gastrocnemius muscles were also dissected. A non-cemented megaprosthesis (Kyocera Modular Limb Salvage system; Kyocera Corporation, Kyoto, Japan) was inserted after wide tumor resection [21]. The patellar component was cemented.

**Figure 2:**
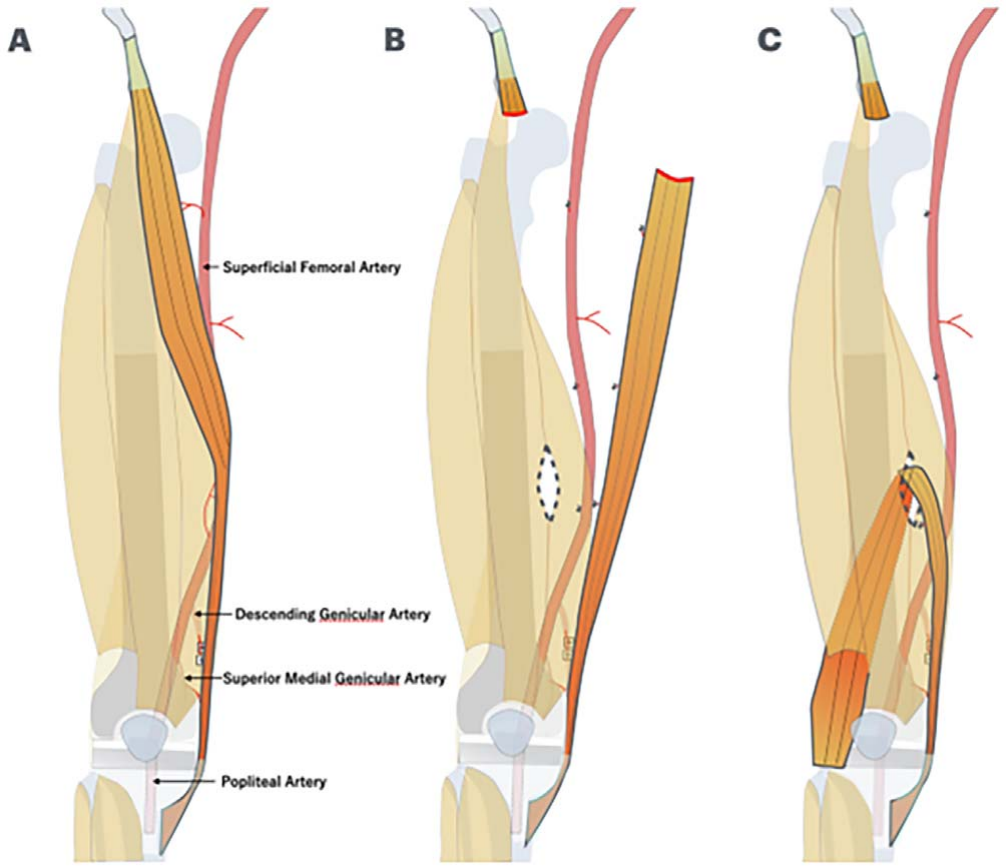
(A) The megaprosthesis of the distal femur was located directly under the skin without any muscle tissue. (B) The proximal portion of the sartorius muscle was fully detached and then the sartorius muscle was dissected at the myotendinous transition in the region near the origin. The intermuscular septum between the sartorius and quadriceps muscles was incised. (C) A hole through which to pass the sartorius muscle flap was created, and the sartorius muscle flap was guided to the lateral thigh to prevent it from twisting between the quadriceps muscle and the femur.

Although primary closure of the wound was possible, the lateral side of the megaprosthesis was covered with a sartorius muscle flap using the distal vascular pedicles because the megaprosthesis was located directly under the skin without any muscle tissue (Figure 2). A skin incision was made in the direction of the sartorius muscle fibers on the ipsilateral side of the sartorius muscle flap elevation. The pivot point was chosen so that the distal two vascular pedicles of the sartorius muscle flap could be retained. To enable the folding of the sartorius muscle toward the lateral side of the knee joint with adequate room, the proximal portion of the sartorius muscle was fully detached and then the sartorius muscle was dissected at the myotendinous transition in the region near the origin. The intermuscular septum between the sartorius and quadriceps muscles was incised. A hole through which to pass the sartorius muscle flap was created, and the sartorius muscle flap was guided to the lateral thigh to prevent it from twisting between the quadriceps muscle and the femur. The sartorius muscle flap was carefully detached until it could no longer be separated by flexing and extending the knee joint. If deemed insufficient, further detachment was performed and finally sutured to the surrounding soft tissue.

The surgical field was thoroughly washed, hemostasis was confirmed, a drain was placed, and the operation was completed with suturing of the fascia, subcutis, and epidermis. The patient underwent 2 weeks of splint immobilization of the affected limb to limit knee flexion and extension movements. At 2 weeks postoperatively, the splint was removed and a knee brace was placed, and the patient began unassisted ambulation. The patient gradually began weight-bearing gait at 4 weeks postoperatively and was allowed to walk with full weight-bearing at 8 weeks postoperatively.

This study was conducted according to the guidelines of the Declaration of Helsinki and approved by our institutional review board (protocol code 2833). The requirement for written consent from the participants was waived because an opt-out process was used and the study was retrospective.

## Results

**Figure 3:**
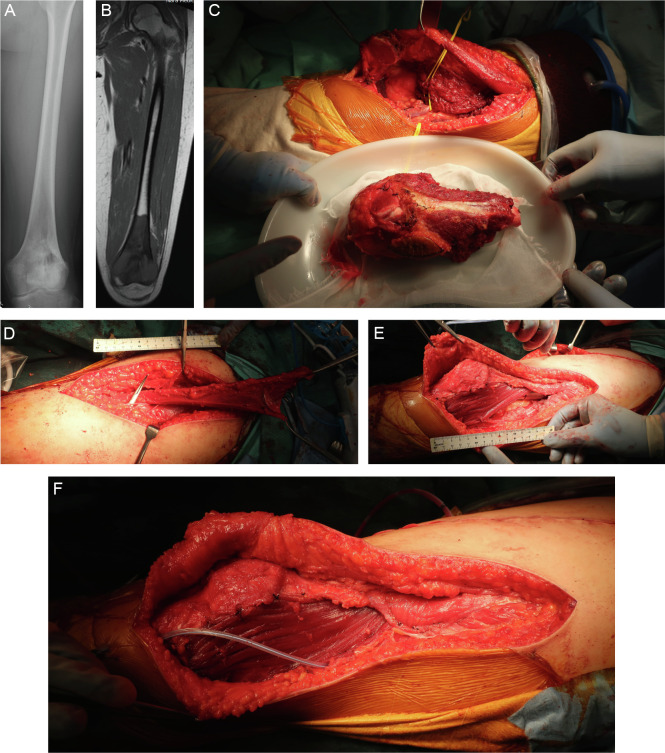
Case 1: 15-year-old female patient. (A, B) An incisional biopsy was performed from the lateral side of the distal femur, and a conventional osteosarcoma in the distal part of the left femur was diagnosed. No distant metastasis was found at the time of the initial diagnosis. Preoperative chemotherapy (methotrexate + doxorubicin + cisplatin regimen) was administered. A preoperative computed tomography scan of the chest revealed multiple lung metastases. (C–F) Wide resection including the skin around the biopsy site, reconstruction with a megaprosthesis, and sartorius muscle flap coverage were performed. After the surgery, the lung metastases were inoperable, and ifosfamide + etoposide, pazopanib, and gemcitabine + docetaxel were administered. However, the lung metastasis could not be controlled, and the patient died 1 year 7 months after the diagnosis.

**Figure 4:**
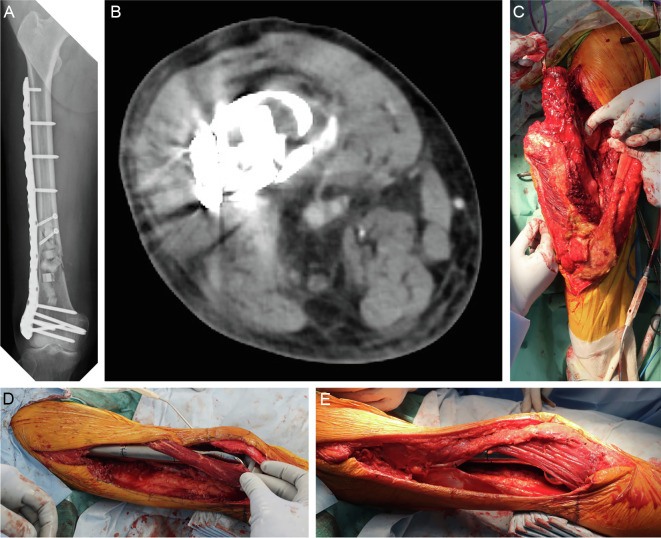
Case 2: 74-year-old man. The patient experienced pain in the distal right femur, and a needle biopsy was performed based on suspicion of an enchondroma or atypical cartilaginous tumor as shown by X-ray and magnetic resonance imaging. The pathological examination also showed findings consistent with an enchondroma or atypical cartilaginous tumor, and the patient was followed up with imaging. Two years after the biopsy, the pain recurred and a pathological fracture developed. (A, B) The fracture site was identified by a lateral approach, and the tumor was curetted using a high-speed burr and cauterized with an argon beam coagulator. The resulting cavity was filled with artificial bone, and the fracture was fixed with a locking plate. Based on the pathological examination of the curettage specimen, a diagnosis of dedifferentiated chondrosarcoma was made. (C–E) Additional wide resection was performed with a 3-cm margin around the previous skin incision, and reconstruction with a megaprosthesis and sartorius muscle flap coverage was performed. Postoperative adjuvant radiation therapy of 66 Gy in 33 fractions was administered. Two months after the additional wide resection, multiple lung and bone metastases were found, and the patient died 6 months after the diagnosis of dedifferentiated chondrosarcoma.

**Figure 5:**
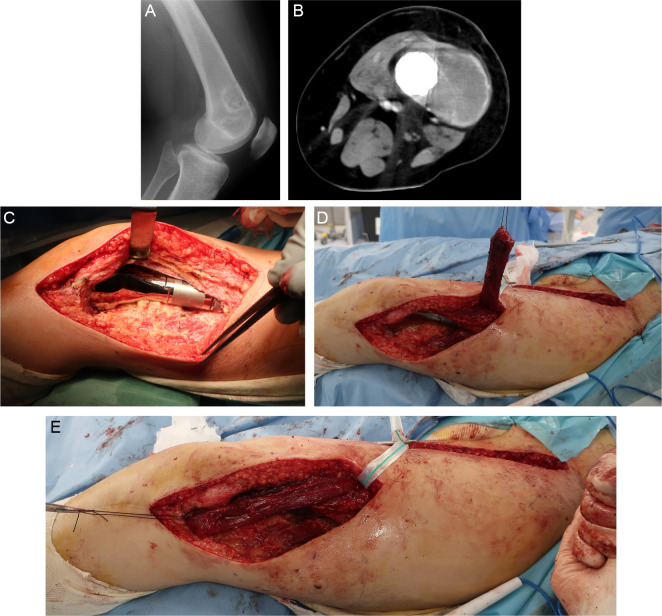
Case 3: 58-year-old woman. (A) An incisional biopsy of the distal femur was performed using a lateral approach, and bone metastasis of uterine leiomyosarcoma was diagnosed. The skin around the biopsy, a part of the vastus lateralis muscle, and the vastus intermedius muscle were resected along with the bone tumor. Reconstruction was then performed with a megaprosthesis. Based on the pathological examination of the surgical specimen, conventional osteosarcoma was diagnosed, and postoperative chemotherapy (methotrexate + doxorubicin + cisplatin regimen) was administered. (B) Three years after surgery, a mass was found on the distal lateral aspect of the left thigh; this was diagnosed as local recurrence by needle biopsy. (C–E) Wide resection of the tumor including the vastus lateralis muscle and the iliotibial tract was performed, and the megaprosthesis was covered with a sartorius muscle flap. The patient had no evidence of disease 5 years 6 months after the diagnosis of osteosarcoma.

The patients’ mean age was 49 years (range, 15–74 years). The histological tumor type was osteosarcoma in two patients and dedifferentiated chondrosarcoma in one patient. The average femoral resection length was 19 cm (range, 14–29 cm), and the average percentage of the total femoral length resected was 45% (range, 35%–63%). The average size of the soft tissue defect on the lateral thigh was 6 × 13 cm, the average time required to elevate the sartorius muscle flap was 100 min, and the average implant coverage was 93%. The surgical margin was R0 in two patients and R1 in one patient. One patient received preoperative and postoperative chemotherapy. No patients received preoperative radiation therapy, and one patient received postoperative radiation therapy. The average postoperative follow-up period was 35 months, during which no patients developed complications such as postoperative infection, skin necrosis, or nerve palsy. Two patients died of their tumors and one patient showed no evidence of disease (Tables 1–3, Figures 3–5).

**Table 1:** Patient characteristics.

Case	Age (years)	Sex	Histology	Biopsy method	Tumor size (cm)	Pathologic fracture	Approach	Knee joint	Bone resection length (cm), percentage of total femoral length	Resected muscle	Defect size (cm)	Implant coverage percentage
1	15	Female	Conventional osteosarcoma	Open	7×6×10	No	Lateral	Extra-articular resection	15, 38%	Vastus medialis, lateralis, intermedius	14×6	100%
2	74	Male	Atypical cartilaginous tumor → Dedifferentiated chondrosarcoma	Unplanned surgery (curettage and plate fixation)	4×3×11	Yes	Lateral	Extra-articular resection	29, 63%	Vastus medialis, lateralis, intermedius, iliotibial tract	14×6	80%
3	58	Female	Local recurrence of conventional osteosarcoma in the vastus lateralis	Needle	4×4.5×8.5	No	Lateral	Extra-articular resection (initial surgery)	14, 35% (initial surgery)	Vastus lateralis, iliotibial tract	11×6	100%

**Table 2:** Surgical details.

Case	Surgical time (min)	Time required for flap (min)	Bleeding amount (mL)	Surgical margin	Preoperative CHT	Postoperative CHT	Preoperative RT	Postoperative RT
1	450	120	300	R0	MAP	IE, pazopanib, GEM/DOC	No	No
2	410	90	380	R1	No	No	No	Yes
3	150	90	23	R0	No (initial surgery)	MAP (initial surgery)	No	No

CHT, chemotherapy; RT, radiotherapy; MAP, methotrexate + doxorubicin + cisplatin; IE, ifosfamide + etoposide; GEM/DOC, gemcitabine + docetaxel

**Table 3:** Patients’ clinical details.

Case	Smoking	DM	BMI (kg/m^2^)	Hb (g/dL)	Alb (g/dL)	Comorbidities	Surgical complications	Local recurrence (months)	Distant metastasis (months)	Outcome	Follow-up from diagnosis (months)
1	No	No	19.9	12.1	4.5	No	No	No	4, lung	DOD	20
2	Yes	Yes	21.0	13.4	4.2	Hypertension	No	4	4, lung and bone	DOD	21
3	No	No	24.6	14.9	4.9	Uterine fibroids	No	50	64, pubis	NED	65

DM, diabetes mellitus; BMI, body mass index; Hb, hemoglobin; Alb, albumin; DOD, death of disease, NED, no evidence of disease

## Discussion

To prevent infection after limb-sparing surgery for primary malignant bone tumors, it is important to cover the megaprosthesis with muscle tissue that has rich blood flow [13, 14]. Although coverage with a lateral gastrocnemius flap has been reported in cases of combined resection of the vastus lateralis and vastus intermedius during distal femoral replacement [12, 15], a high risk of peroneal nerve palsy (44%) due to passage of the muscle flap near the fibular head has been reported [12]. We have herein described three patients with primary malignant bone tumors of the distal femur who underwent wide tumor resection (including the vastus lateralis and vastus intermedius) followed by reconstruction with a megaprosthesis and coverage of the lateral side of the megaprosthesis with a sartorius muscle flap. Implant coverage was possible, and no skin necrosis, deep infection, or nerve palsy was observed. Because the sartorius muscle is located in a superficial area and is easy to elevate in the supine position with minimal functional loss after harvesting and a minimal risk of nerve palsy [20], it can be advocated as the first choice for coverage of soft tissue defects on the lateral side of distal femoral replacements.

One systematic review showed that the risk of infection in cases of distal femoral replacement was 8.5% [22]. Reported risk factors for periprosthetic infection of a megaprosthesis include inadequate soft tissue coverage, prolonged and repeated surgery, immune suppression, low hemoglobin and albumin concentrations, chemotherapy, radiation therapy, extra-articular resection, hematoma formation, and comorbidities such as diabetes [10, 23–27].

Morii et al. [13] reported that skin necrosis and superficial infection were risk factors for deep infection after reconstruction with megaprostheses around the knee. In the distal femur, three or four quadriceps muscle resections were associated with significantly higher frequencies of skin necrosis, superficial infection, and deep infection than one or two resections [13]. Kawai et al. [14] reported that 18 of 82 patients (22%) who underwent distal femoral replacement developed wound complications. Among these 82 patients, 6 of 54 patients (11.1%) in whom one or more quadriceps muscles could be preserved developed skin necrosis or infection, whereas 10 of 28 patients (35.7%) in whom all quadriceps muscles were removed developed skin necrosis or infection (*P* = 0.016) [14]. Skin necrosis is caused by cutting of the perforator due to extensive subcutaneous detachment as well as poor vascular distribution in the skin. Without soft tissue coverage, skin necrosis increases the risk of deep infection and worsens the patient’s prognosis [14, 15]. If much of the quadriceps muscle is resected, the megaprosthesis must be covered with a muscle flap [14, 15]. The use of a rotational muscle flap or a vascularized free muscle flap should be considered for this purpose [14, 15]. Extra-articular resection requires more extensive soft tissue dissection than intra-articular resection, making it more difficult to cover the megaprosthesis with muscle [28]. Therefore, implant coverage with a muscle flap should be considered in patients requiring extra-articular resection. In fact, Myers et al. [29] reported that the infection rate before the introduction of a gastrocnemius flap for proximal tibial replacement was 31% but only 14% after the introduction.

Several options are available for the reconstruction of soft tissue defects around the knee joint, including local skin, muscle, and myocutaneous flaps. For large defects, reconstruction with a free muscle flap is necessary [19]. Free muscle flaps are useful, but they require microsurgical vascular anastomosis techniques and postoperative hemodynamic problems have been reported in approximately 5% of cases [19]. When flaps are combined with reconstruction using a megaprosthesis, the risk of amputation in case of failure must always be kept in mind [7]. By contrast, a pedicled muscle flap has fewer postoperative problems and is easier to combine with reconstruction using a megaprosthesis [30]. A pedicled medial gastrocnemius flap is useful for reconstructing soft tissue defects of the medial knee joint. For reconstruction of soft tissue defects of the lateral knee joint, the use of a lateral gastrocnemius flap, gracilis muscle flap, or peroneus longus muscle flap has been reported [19]. The lateral gastrocnemius muscle belly is smaller than the medial one, and lateral gastrocnemius muscle flaps are associated with a risk of common peroneal nerve injury [12, 19]. The gracilis muscle flap and peroneus longus muscle flap are small and can only be adapted to small defects [19]. In the present study, reconstruction with a sartorius muscle flap utilizing distal vascular pedicles allowed coverage of 93% of the area of the megaprosthesis and did not result in skin necrosis, deep infection, or nerve injury.

The use of the sartorius muscle flap was first reported in 1948 for coverage of femoral artery exposure after inguinal lymph node dissection [31]. Numerous anatomical studies of the sartorius muscle flap have been performed. The vascular distribution of the sartorius muscle flap was long thought to be Mathes and Nahai type IV, but a recent anatomical study by Mojalla et al. [18] revealed a type III vascular distribution. The majority (80–90%) of the sartorius muscle is nourished exclusively by major vascular pedicles, either proximal or distal [32, 33], and use of the sartorius muscle flap with distal vascular pedicles has been reported for soft tissue defects after trauma [17]. The major distal vascular pedicle of the sartorius muscle flap is reportedly located 35 cm from the ASIS [17]. Similar studies on the location of the major distal vascular pedicle have shown that it is positioned 34 to 43 cm from the ASIS [18] and 10 cm from the pes anserinus [34]. The distal vascular pedicles can originate from the superficial femoral artery, descending genicular artery, popliteal artery, and superior medial genicular artery [18, 35].

When covering soft tissue defects on the lateral side of the knee joint, the pivot point is proximal to the distal vascular pedicle. Therefore, if the location of the distal vascular pedicles can be confirmed preoperatively, there is no need to expose the vascular pedicles during muscle flap elevation; this markedly reduces the risk of vascular pedicle injury [18, 35]. In addition, the lateral gastrocnemius flap, gracilis muscle flap, and peroneus longus muscle flap require rotation of the lower extremity for elevation, whereas the sartorius muscle flap can be easily elevated in the supine position [19]. Regarding elevation of the sartorius muscle flap, clinical studies suggest that the maximum safe arc of rotation is approximately 130° [17]. However, when the flap is used for lateral knee joint coverage, this angle is unlikely to be exceeded even if two or more distal vascular pedicles are preserved [18, 35]. The sartorius muscle flap is an elongated muscle in the longitudinal direction, but its relative thickness allows it to be extended perpendicular to the direction of muscle fibers, making it possible to cover a soft tissue defect of approximately 6 cm in width [18, 35]. Placement of a distally based sartorius muscle flap is a simple procedure with minimal functional loss after harvesting, and it is a useful option for soft tissue reconstruction of the lateral knee joint following distal femoral replacement for malignant bone tumors.

This study had two main limitations. First, it was a retrospective study involving a small number of patients. Second, this study had no control group of patients who underwent soft tissue reconstruction using a lateral gastrocnemius flap. However, there is no major nerve near the sartorius muscle that can cause severe movement disorders after placement of the sartorius muscle flap, and the risk of severe movement disorders such as peroneal nerve palsy is thus extremely low [18, 35]. It is necessary to conduct a multicenter collaborative study, analyze a large number of patients, and determine which muscle flap is optimal for coverage of soft tissue defects in distal femoral replacement for bone tumors.

## Conclusions

Placement of a sartorius muscle flap using distal vascular pedicles is an easy surgical technique with minimal functional loss after harvesting and a low risk of nerve injury. It may replace the lateral gastrocnemius flap as the first choice for the reconstruction of soft tissue defects on the lateral thigh in patients undergoing distal femoral replacements for treatment of bone tumors.

## Data Availability

The datasets generated, analyzed, or both during the present study are not publicly available because of privacy issues but are available from the corresponding author upon reasonable request.
